# Effect of Pharmacy Student Peer Supervision on the Accuracy of Admission Medication Reconciliation: Prospective Pre-Post Observational Study

**DOI:** 10.2196/77486

**Published:** 2026-03-09

**Authors:** Basma Ammor, Morgane Masse, Anne Toulemonde, Laurine Cadart, Jean-Baptiste Beuscart, Marc Lambert, Pascal Odou, Bertrand Décaudin

**Affiliations:** 1 CHU Lille, Institut de Pharmacie, Lille, F-59000, France, 33 0320964030; 2 Univ Lille, CHU Lille, ULR 7365-GRITA-Groupe de Recherche sur les formes Injectables et les Technologies Associées, Lille, F-59000, France; 3 CHU Lille, Service de Biostatistiques, Lille, F-59000, France; 4 Univ Lille, CHU Lille, ULR 2694 - METRICS : Évaluation des Technologies de Santé et des Pratiques Médicales, Lille, F-59000, France; 5 Service de médecine interne, centre national de référence maladies systémiques et auto-immunes rares (sclérodermie systémique), CHU de Lille, UFR de médecine, Université de Lille, Lille, F-59000, France

**Keywords:** clinical pharmacy, admission medication reconciliation, peer validation, pharmacy students, supervision

## Abstract

**Background:**

Although medication reconciliation is known to reduce the frequency of medication errors, its practical implementation can be challenging in several respects. In our institution, pharmacy students perform medication reconciliations at admission under the supervision of a pharmacist or pharmacy resident.

**Objective:**

The objective of the study was to evaluate the impact of peer supervision (ie, the supervision by a pharmacy student of a medication reconciliation performed by another pharmacy student) on the accuracy and efficiency of admission medication reconciliations.

**Methods:**

A prospective, single-center, observational study was conducted in 2 clinical departments at Lille University Medical Center (Lille, France). Initially, organizational procedures were defined and a checklist for reconciliation supervision was developed. A baseline (reference) period without peer supervision was compared with an implementation period with peer supervision.

**Results:**

A total of 317 medication reconciliations were conducted: 102 (32.2%) without supervision and 215 (67.8%) with supervision by a pharmacy student. Peer supervision reduced the pharmacist time required for this task by 52%; the mean time decreased from 23 (SD 11) minutes to 11 (SD 6) minutes. Furthermore, peer supervision was associated with a decrease in the number of errors made by students (from 1.5 to 0.9 per reconciliation) and detected by pharmacists during reconciliation validation.

**Conclusions:**

Student peer validation appears to be an innovative, strategic method for optimizing medication reconciliations, freeing up pharmacist time, and leveraging the skills of pharmacy students.

## Introduction

Admission medication reconciliation plays a crucial role in the prevention and interception of medication errors [1,2]. It ensures the accurate and comprehensive transfer of information about patients’ home medications among health care professionals [3]. Admission medication reconciliation also addresses other objectives, including continuity of treatment, diagnosis of drug-related iatrogenesis, and establishment of a reliable reference dataset for subsequent medication reviews.

Patient admission is a critical moment for medication safety: the risk of prescription errors is 70% higher on admission than during the rest of the hospital stay [4]. To address this problem, admission medication reconciliation is crucial for the improvement of patient safety and care quality. This practice is now essential in modern health care. With a view to maximizing the benefits of admission medication reconciliation and reducing medication-related errors, our institution (Lille University Medical Center, Lille, France) is now considering the expansion of this reconciliation technique from high-risk patients only to a broader range of patients [5].

The implementation of clinical pharmacy activities requires changes in organizational practices as a function of available human resources. In our institution, about 50 pharmacy students are assigned to clinical departments. Some of these students conducted medication reconciliations by preparing “best possible medication histories” (BPMHs) [6]. These histories were then compared with admission prescriptions under the supervision of a pharmacist or pharmacy resident [7]. All the pharmacy students attended a standardized program of practical and knowledge-based training sessions developed by 4 of our clinical pharmacists. Each student was then qualified by the supervising pharmacist of the clinical service using a validated checklist of criteria.

Medication reconciliation is increasingly recognized as a critical component of patient safety and is now integrated into care pathways in many health care systems. In France, its implementation is included as a certification criterion by the French National Authority for Health (*Haute Autorité de Santé*, HAS), particularly for high-risk patient groups, such as those in geriatrics and oncology. Despite its recognized importance, medication reconciliation remains challenging in hospital settings, primarily due to the significant time investment required from pharmacists, which competes with their other responsibilities. Its successful implementation depends on both the commitment of health care professionals and the resources allocated, factors that are often limited in practice [8,9]. There are various strategies for optimizing hospital resources, especially when reconciliation involves a number of different health care stakeholders (eg, pharmacy technicians and nurses) [10,11]. The BPMHs drawn up by pharmacy students and pharmacy technicians as part of the medication reconciliation process are accurate and efficient, reduce costs, and provide support for other health care professionals [12]. However, some errors persist, due to the students’ limited clinical experience and the relatively short duration of their placements—typically up to 3 months and sometimes on a part-time basis. Therefore, oversight by experienced pharmacists remains essential to maintain the quality and safety of the reconciliation process, given that these individuals are still in training. In our acute geriatric units (AGUs) and internal medicine departments (IMDs), medication reconciliation is particularly time- and resource-intensive; hence, we are exploring organizational changes that can address constraints on pharmacist time. One of these changes might be the introduction of student supervisors as a means of supporting pharmacy residents and pharmacists.

We hypothesized that incorporating peer review, a process commonly used in scientific research for critical evaluation [13], into medication reconciliation could both optimize pharmacist time and maintain high-quality practices. Additionally, supervision and peer review may provide pharmacy students with valuable opportunities to develop essential professional skills, including effective communication, priority management, teamwork, and cohesion.

The objective of this study was to evaluate the impact of a peer supervision process among pharmacy students on the accuracy and efficiency of admission medication reconciliations.

## Methods

### Study Design

This was a prospective, single-center, observational study conducted at Lille University Medical Center in 2 phases in an AGU and an IMD. In the initial, qualitative phase, we developed an organizational model. In the second phase, we evaluated this organizational model by collecting data on admission medication reconciliations for patients aged 65 years and older.

Medication reconciliation was performed in accordance with the recommendations of Haute Autorité de Santé, which defines four essential steps in its implementation: (1) collection of information on medications currently taken or intended to be taken by the patient, (2) development of a BPMH synthesizing this information, (3) validation of the BPMH, and (4) sharing and use of the BPMH within the care team [10]. The medication reconciliation process was conducted under the responsibility of each clinical pharmacist who was a member of the multidisciplinary team involved in the patient’s care. This process did not include medication prescribing by the pharmacist but was based on communication and collaboration with the physician responsible for the patient’s follow-up.

To ensure that reconciliations were comprehensively reviewed by supervisors, we considered various tools for guiding the students. Discussions between the initiating investigators thus led to the development of a checklist for the student supervisor. The supervisory checklist was developed through a multistep, consensus-building process [14] (MultimediaAppendices 1^4^). First, 2 investigators created a draft version of the checklist and shared it with the reference pharmacists involved in medication reconciliation. The latter evaluated each item, provided feedback, and suggested improvements. The checklist was revised accordingly and sent back to the reference pharmacists for further assessment. This iterative process continued until a consensus on the checklist’s content was reached.

The study was conducted from December 2023 to May 2024 in an AGU and an IMD at Lille University Medical Center. The evaluation period comprised a baseline phase from December 2023 to February 2024 (during which medication reconciliation data were collected without supervision and without a checklist) and an intervention phase. Although consensus on the final checklist was achieved in April 2024, an initial version was used for supervision in February and March 2024. The evaluation involved a reorganization that allowed trained volunteer students to review, modify, and validate their peers’ admission medication reconciliations in collaboration with pharmacists and pharmacy residents. The BPMHs and the medical prescriptions at admission were recorded in our medical center’s electronic patient record system (Sillage, version 22.1; MipihSIB).

**Figure 1.**
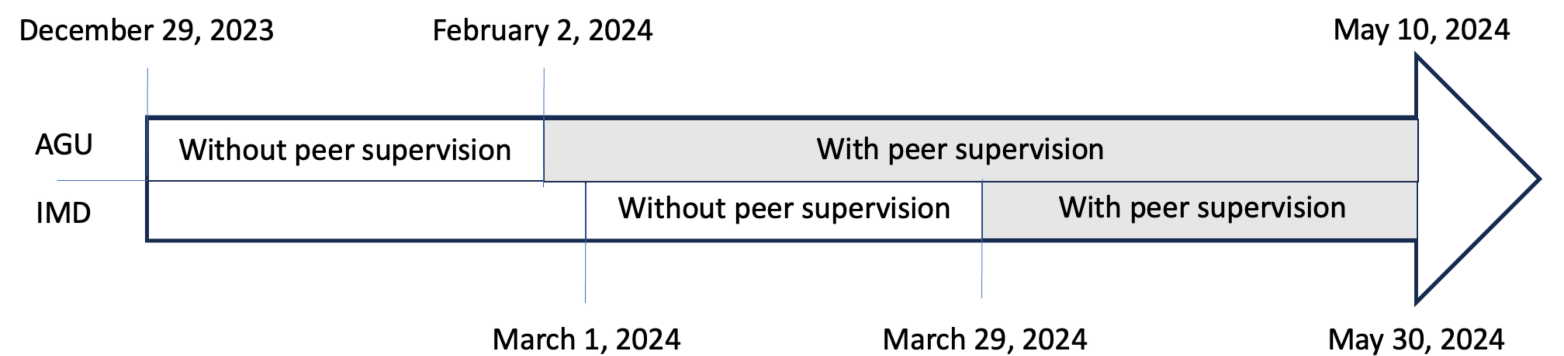
Study timeline by department. AGU: acute geriatric unit; IMD: internal medicine department.

For each department, the evaluation was carried out over 2 periods (Figure 1). The first period served as a baseline: admission medication reconciliations performed by pharmacy students were supervised solely by pharmacy residents or pharmacists. The second (intervention) period corresponded to student supervision of admission medication reconciliations conducted by other pharmacy students. The supervising students were trained in 2 stages. Initially, a clinical pharmacist with postgraduate specialty training and 7 years of experience or a final-year pharmacy resident in clinical pharmacy supervised an admission medication reconciliation and demonstrated the method to the student. Once the student had learned the method, they supervised a reconciliation by another student but under the observation of the clinical pharmacist or the final-year pharmacy resident in clinical pharmacy. If the pharmacist validated the student’s skills, the latter was allowed to supervise another student’s admission medication reconciliations alone.

### Variables

The study data were collected by 2 investigators (a clinical pharmacist in the IMD and a final-year pharmacy resident in the AGU) using a standardized collection form. For each reconciliation, the following data were recorded. First, we recorded the total time taken by the student to complete the medication reconciliation [15]. In both phases of the study, this duration included the time spent collecting and checking the patient’s medication information for a BPMH, comparing the BPMH with the medicines prescribed at admission, and identifying and rectifying any discrepancies [7]. The BPMH integrates multiple information sources, prioritized according to their reliability, including primary objective data (outpatient pharmacy dispensing records and electronic prescriptions), interviews with community pharmacists, telephone contact with the primary care physician, prior medical documentation of treatment changes, patient or caregiver interviews, and hospital medical records. Patient-reported discrepancies that cannot be independently verified are flagged. Reconciliation accuracy is assessed by comparing the BPMH with clinical judgment and current prescriptions: clinically justified discrepancies (eg, temporary withholding of anticoagulant therapy due to bleeding risk) are considered appropriate when clearly documented and communicated, whereas unintentional discrepancies (such as omissions, duplications, or dosing errors) are classified as medication errors requiring resolution. Second, we recorded the time taken by the pharmacist to validate the medication reconciliation (after the latter had been supervised by a student, if applicable), up until the discussion with the prescriber. Third, we recorded the errors identified and corrected by the student supervisor and/or pharmacist, including the number, type, and medications involved. An “error” was defined as an omission, unjustified addition, or misinterpretation of an element in the student’s observation that contradicted the instructions received during the training or a specific item of the validation checklist. All identified errors were analyzed. Fourth, we recorded the total number of medications per BPMH. Fifth, we recorded the adverse drug event risk score developed by Trivalle et al [16], which has been validated in patients aged 65 years and older in France. The score is derived from 3 risk factors: polypharmacy (≥7 medications), antipsychotic treatment, and recent anticoagulant exposure within the previous 3 months. Sixth, we recorded the initials of the student supervisor and the validating pharmacist.

The number of errors identified and corrected by pharmacists, as well as the time required for pharmacists to validate medication reconciliations, was assessed in 2 independent samples before and after the implementation of student supervision. This approach enabled us to estimate the impact of student oversight on the quality and efficiency of admission medication reconciliations performed by peers. No threshold for an acceptable number of errors was defined; all errors were recorded, whether identified by the student supervisor or the supervising pharmacist, without distinguishing between the 2 sources.

### Statistical Analysis

The data were entered into computer spreadsheets (Microsoft Excel). Qualitative variables were described as the frequency (percentage). Quantitative variables were described as the mean (SD) or, when the data were not normally distributed, the median (IQR). The normality of distribution was assessed both graphically and with the Shapiro-Wilk test.

The 2 periods (baseline and intervention) were compared using 2-tailed Student *t* test or, when the data were not normally distributed, the Mann-Whitney *U* test. The comparison included all medication reconciliations performed without peer supervision (baseline phase) and all peer-supervised medication reconciliations (intervention phase). Reconciliations performed by student supervisors were not included because they did not involve supervision, as only 1 supervisor was available per week.

The number of errors before and after the introduction of student supervision was analyzed using a generalized linear regression model for count data (negative binomial distribution with log link function), adjusted for confounding factors (the number of medications per BPMH, the adverse drug event prediction score, and time taken by the student to perform the reconciliation). An adjusted rate ratio (RR) with 95% CI was reported as a measure of association. The time taken by the pharmacist to correct and validate the reconciliation before and after the introduction of student supervision was compared by applying the same method.

Factors associated with the number of errors found in each reconciliation by the pharmacist were identified in a generalized linear regression model for count data (negative binomial distribution with a log link function). Again, the RR (95% CI) was calculated as a measure of association. Factors associated with the time taken by the pharmacist to correct and validate the reconciliation were identified by applying the same method.

The association between the number of errors and the time taken by the pharmacist was assessed by calculating the Spearman correlation coefficient (*ρ*).

The threshold for statistical significance was set at *P*<.05. All statistical analyses were performed using SAS software (version 9.4; SAS Institute Inc).

### Ethical Considerations

No personal data (medical or otherwise) of the patients involved in the reconciliations were collected. In line with French legislation, neither informed consent nor approval by an institutional review board was required or sought. The study database was registered with the data protection officer of Lille University Hospital under the terms of the MR-004 French national reference methodology [17] on the processing of personal data for study, evaluation, or research purposes not involving human subjects.

## Results

### Overview

The evaluation phase involved various pharmacists and pharmacy students. The first part involved 2 pharmacists, and the second part involved 15 students (including n=8, 53.3% supervisors), 3 pharmacists, and 2 pharmacy residents. Data on 317 admission medication reconciliations were collected: 102 (32.2%) reconciliations were not peer supervised (n=31, 9.8% in the AGU and n=71, 22.4% in the IMD), and 215 (67.8%) were peer supervised (n=191, 60.3% in the AGU and n=24, 7.6% in the IMD). A rotation system had been set up in the AGU: of the 4 pharmacy students involved, 1 supervised the reconciliations for 2 weeks at a time, and the remaining 3 performed the reconciliations. In the IMD, a student supervised the other 2 students.

### The Medication Profile of the Patients Included in the Study

The mean number of medications per BPMH was higher during the student-supervised period than during the nonsupervised period (9.6, SD 3.8 vs 8.6, SD 4 medications, respectively), whereas the mean total time needed for reconciliation was shorter (138, SD 42 min vs 174, SD 96 min, respectively; Table 1). The adverse drug event risk score was similar in the 2 periods.

**Table 1.** Comparison of the reference and implementation phases regarding patient medication profiles and the duration of reconciliations performed by students.

	Without peer supervision (n=102)	With peer supervision (n=215)	*P* value
Adverse drug event risk score, median (IQR)	3.0 (0-6)	4.0 (1-6)	.10
Total number of medications in the best possible medication history, mean (SD)	8.6 (4)	9.6 (3.8)	.047
Total time taken by the student to complete the reconciliation (min), mean (SD)	174 (96)	138 (42)	<.001

### Factors Influencing the Time Taken by the Pharmacist to Validate a Reconciliation

The time taken by the pharmacist was significantly associated with the number of medications per BPMH (RR=1.03, 95% CI 1.01‐1.04; *P*=.001). However, this time was not influenced by the adverse drug event prediction score (RR=1.02, 95% CI 0.99‐1.04; *P*=.05) or the time taken by the student to perform the reconciliation (RR=1.02, 95% CI 0.96‐1.08; *P*=.06).

### Factors Influencing the Number of Errors Corrected by the Pharmacist

The number of errors corrected by the pharmacist was significantly associated with the number of medications per BPMH (RR=1.09, 95% CI 1.05‐1.12; *P*<.001), the time taken by the student to perform a reconciliation (RR=1.19, 95% CI 1.06‐1.33; *P*=.004), and the adverse drug event prediction (RR=1.10, 95% CI 1.05‐1.14; *P*<.001).

### Impact of Peer Supervision

Our results showed that the number of errors was smaller and the total pharmacist time was shorter during the student-supervised period than during the nonsupervised period (Table 2). The mean time taken by the pharmacist to rectify errors in reconciliation was significantly shorter during the student-supervised period (mean 11, SD 6 minutes) than during the nonsupervised period (mean 23, SD 11 minutes); this corresponded to a time saving of 53% (RR=0.47, 95% CI 0.42‐0.52; *P*<.001). Moreover, the number of errors corrected by the pharmacist fell from 1.5 to 0.9 (a 36% decrease; RR=0.64, 95% CI 0.49‐0.83; *P*<.001). For the study period as a whole (N=317 admission medication reconciliations), we observed a significant linear correlation between the time taken by the pharmacist and the number of errors rectified (*ρ*=0.45, 95% CI 0.38‐0.55; *P*<.001).

**Table 2.** Impact of peer supervision on the number of errors corrected and the pharmacist validation time.

	Without peer supervision (n=102), mean (SD)	With peer supervision (n=215), mean (SD)	Rate ratio (95% CI)	*P* value
Time taken to validate the reconciliation (minutes)	22.5 (10.9)	11.4 (6.08)	0.47 (0.42‐0.52)	<.001
Number of errors corrected	1.5 (1.51)	0.9 (1.24)	0.64 (0.49‐0.83)	<.001

### Analysis of Errors

We identified 143 errors corrected by student supervisors or pharmacists (Multimedia Appendix 5). Not all errors were described in detail. The most frequent error (accounting for n=25, 17.5% of all errors) was the omission of the precise administration frequency for as-needed (ie, on-demand) medications. The second most frequent error (n=13, 9.1%) was the misinterpretation of differences between the patient’s medication history and the admission prescriptions.

The analysis of reconciliation errors also enabled us to identify the most frequently involved drug classes: other analgesics and antipyretics were involved in 28 (19.6%) errors, followed by antihypertensives (n=18, 12.6%; Multimedia Appendix 6).

## Discussion

### Principal Findings

Peer validation of admission medication reconciliations by pharmacy students proved to be effective in optimizing the pharmacists’ working time; the average pharmacist time per reconciliation decreased from 23 minutes to 11 minutes, and the average number of errors made by the student conducting the reconciliation fell from 1.5 to 0.9. However, these reconciliation time savings must be balanced against the time required to train the student supervisors, that is, 2 to 4 half-days. We observed a significant linear correlation between the time spent by pharmacists and the number of errors rectified (*P*<.001), which confirmed the validity of our data.

In France, the current regulations do not specify how admission medication reconciliations should be validated. Here, we chose to have all student-conducted and student-supervised reconciliations validated by a pharmacist or a resident to ensure the quality of the work performed. Accordingly, the pharmacy students entered their reconciliation reports into our medical center’s electronic patient record system so that the validated reconciliations could be consulted by the prescribers. According to the French Society of Clinical Pharmacy (Société française de pharmacie clinique), the pharmacist is the guarantor of the reconciliation process [18] and the validation of certain steps in this process [15].

Peer evaluation in pharmacy education is a valuable pedagogical tool that helps students develop key skills such as communication, teamwork, priority management, and critical thinking [19]. It fosters accountability, self-reflection, empathy, and cultural sensitivity within a supportive, student-centered environment. Peer learning also enhances satisfaction for both students and supervisors, promotes lifelong learning, and is cost-effective [20]. Although commonly used in research and initial training, peer evaluation is less documented in hospital pharmacy settings [21-23]. However, studies show that peer mentoring improves academic success [24] and practical skills. Peer supervision encourages open feedback [25], motivation, and confidence by creating a less hierarchical and collaborative learning atmosphere [26]. However, successful implementation relies on a culture of transparency and mutual trust. Peer supervision may also improve the quality of patient care and can help pharmacy students to acquire skills that will be essential in their careers.

With a view to improving the student supervision of admission medication reconciliations, we developed a checklist. This type of tool is known to be effective because it ensures that no details are overlooked, saves time (by providing a written reference instead of relying on memory), and enhances overall organization. Checklists are widely used in the medical field in general and operating rooms in particular; the cross-checking of essential criteria before, during, and after each operation helps increase levels of safety and efficiency [27].

It has been reported that the empowerment of students with medication reconciliation tasks positively influences their perception of future responsibilities and helps them develop field-specific, general, and organizational skills [28]. Active participation enhances the students’ perceived level of autonomy [29]. In a questionnaire, the pharmacists stated that they favored student supervision of admission medication reconciliations under certain conditions, such as those with specialty-specific issues and medication switching. Student involvement allows health care institutions to free up pharmacist time and reinforces trust in medication histories [30]. Trust—based on training, evaluations, and qualifications—is crucial for assigning tasks and peer supervision. Key concepts guiding supervising students are professionalism, self-awareness, and communication [31]. Training in medication reconciliation is essential and improves students’ comfort, confidence, and skills [32]. Supervisors require specific training and experience to assess their peers’ work accurately. Learning programs enable students to contribute to continuity of care activities and are crucial in pharmacy faculties for enhancing preparation for practice, adding value to patient care, and benefiting the health care system [33].

Although these positive outcomes were evident, the student supervisors’ impact on admission medication reconciliations might have been underestimated. During the reference phase (ie, with nonsupervised reconciliations), the pharmacy students were well integrated into the team and were actively engaged in discussions with prescribers before validation. This high level of integration might have reduced the number of errors (relative to what might be expected with novice students) and thus might have partly masked the true benefits of supervision. Furthermore, reconciliations performed by student supervisors were not included in the evaluation phase because they did not involve supervision, which explains the lower number of reconciliations. Other limitations of our study should be acknowledged, including an imbalance in recruitment between the 2 departments and the single-center nature of the study, which warrants confirmation of our findings in other organizational contexts.

This research could be extended by an evaluation of the system in other health care departments, a comparison of the clinical pharmacists’ activity indicators with and without student pharmacist supervision, and the measurement of levels of satisfaction among students, pharmacists, and prescribers [34].

Analysis of errors in our geriatric population showed that first-line analgesics were most frequently involved, with the main error being students’ failure to specify the dosing frequency for as-needed medications. It remains unclear whether these errors were due to knowledge gaps or simple oversight, as we did not distinguish between errors made under supervision by more or less experienced supervisors. Further evaluation of pharmacist interventions and the context of these errors would help clarify whether they stem from carelessness on the part of the student or a lack of professional practice-based knowledge.

Supervision develops autonomy and can be viewed as an entrustable professional activity, that is, a specific task that a student can perform independently once competent [35]. This approach balances autonomy against safety, especially in areas such as geriatrics, where there is a high risk of adverse drug events and potentially unfamiliar medication switches. Supervision creates a training pathway toward autonomous practice, although senior verification is still required to ensure care quality and patient safety. According to the literature on medication review analysis, the integration of a checklist into an artificial intelligence process might enhance efficiency, reduce errors, and improve traceability [36]. However, final validation by a pharmacist is still essential for maintaining rigorous standards and high-quality outcomes amid ongoing technological progress.

### Conclusions

A new peer supervision method for admission medication reconciliations by pharmacy students optimized pharmacists’ working time and was successfully integrated into our IMDs and AGUs. This validation process helped identify and correct potential errors or inconsistencies before reconciliations were finalized. Peer supervision reduced errors through the use of a standardized procedure and specific training. By decreasing the number of errors to be corrected, this approach reduced the time that pharmacists spent validating reconciliations. This new organizational model had a positive impact by improving pharmacists’ time management and availability for other activities, while also providing students with substantial educational benefits in medication reconciliation.

## Supplementary material

10.2196/77486 Multimedia Appendix 1 Development of the organizational model.

10.2196/77486 Multimedia Appendix 2 Responses from pharmacists (first questionnaire; Q1) and pharmacy students (second questionnaire; Q2).

10.2196/77486 Multimedia Appendix 3 Votes on validation in various reconciliation scenarios.

10.2196/77486 Multimedia Appendix 4 Student supervisor’s checklist.

10.2196/77486 Multimedia Appendix 5 Type of errors found in reconciliations supervised by students.

10.2196/77486 Multimedia Appendix 6 Drug classes involved in errors in reconciliations supervised by students.

## Abbreviations

AGU: acute geriatric unit
BPMH: best possible medication histories
IMD: internal medicine department
RR: rate ratio

## References

[1] Phatak A, Prusi R, Ward B (2016). Impact of pharmacist involvement in the transitional care of high-risk patients through medication reconciliation, medication education, and postdischarge call-backs (IPITCH Study). J Hosp Med.

[2] Schnipper JL, Kirwin JL, Cotugno MC (2006). Role of pharmacist counseling in preventing adverse drug events after hospitalization. Arch Intern Med.

[3] Allenet B, Juste M, Mouchoux C (2019). De la dispensation au plan pharmaceutique personnalisé: vers un modèle intégratif de pharmacie clinique. Pharm Hosp Clin.

[4] Dornan T, Ashcroft D, Heathfield H (2009). An in depth investigation into causes of prescribing errors by foundation trainees in relation to their medical education. EQUIP study. https://www.gmc-uk.org/cdn/documents/final-report-prevalence-and-causes-of-prescribing-errors_pdf-28935150.pdf.

[5] Belda-Rustarazo S, Cantero-Hinojosa J, Salmeron-García A, González-García L, Cabeza-Barrera J, Galvez J (2015). Medication reconciliation at admission and discharge: an analysis of prevalence and associated risk factors. Int J Clin Pract.

[6] Best possible medication history. NSW Government.

[7] Medication reconciliation. NSW Government.

[8] (2024). La conciliation médicamenteuse: enquête sur son déploiement national. French Ministry of Health.

[9] Baranyai A, Vaniet F, Coussemacq M, Cousein E La conciliation médicamenteuse. Société Française de Médecine d’Urgence.

[10] (2019). Conciliation des traitements médicamenteux – prévenir les erreurs. Haute Autorité de Santé.

[11] Duwez M, Valette A, Foroni L, Allenet B (2019). Implication du préparateur en pharmacie hospitalière dans le déploiement de la conciliation des traitements médicamenteux en France: représentations et engagement des préparateurs. Ann Pharm Fr.

[12] Champion HM, Loosen JA, Kennelty KA (2019). Pharmacy students and pharmacy technicians in medication reconciliation: a review of the current literature. J Pharm Pract.

[13] Ross-Hellauer T (2017). What is open peer review? A systematic review. F1000Res.

[14] Makhmutov R (2021). The Delphi method at a glance. Pflege.

[15] Le Maoût M, Satori D, Chaouch AA, Corsin L, Dalle-Pécal M (2024). Intégration de la conciliation médicamenteuse dans le parcours de soins du patient insuffisant cardiaque âgé. Actual Pharm.

[16] Trivalle C, Burlaud A, Ducimetière P, IMEPAG Group (2011). Risk factors for adverse drug events in hospitalized elderly patients: a geriatric score. Eur Geriatr Med.

[17] (2018). Délibération n° 2018-155 du 3 mai 2018 portant homologation de la méthodologie de référence relative aux traitements de données à caractère personnel mis en œuvre dans le cadre des recherches n’impliquant pas la personne humaine, des études et évaluations dans le domaine de la santé (MR-004). Légifrance.

[18] (2015). SFPC mémo conciliation des traitements médicamenteux. Société Française de Pharmacie Clinique.

[19] Plott AJ, McIntosh T, O’Ferral H, Bennett MC, Taylor S (2021). Impact of early direct patient care introductory pharmacy practice experiences on student pharmacists’ career exploration. Curr Pharm Teach Learn.

[20] Dyar A, Henriksson P, Stenfors T, Lachmann H, Kiessling A (2024). Differences in supervision on peer learning wards: a pilot survey of the supervisor’s perspective. Adv Med Educ Pract.

[21] Stark JE, Cole JL, Barnes LM, Chapman A, Costner M (2022). Implementation of a novel learning experience in scientific writing, publishing, and peer review into a first year pharmacy practice residency. J Am Pharm Assoc (2003).

[22] Le S, Howard ML (2023). Peer evaluation of teaching programs within pharmacy education: a review of the literature. Curr Pharm Teach Learn.

[23] Morbitzer KA, McLaughlin JE, Henson B, Fassett KT, DiVall MV (2022). Current state of and opportunities for enhancing peer evaluation practices across the pharmacy academy. Am J Pharm Educ.

[24] Asal NJ, Provisor EM, Ryu R (2024). Peer mentoring in pharmacy programs: recommendations for implementation based on a review of available literature. Curr Pharm Teach Learn.

[25] (2019). #PeerRevWk19 is almost here!. X (formerly Twitter).

[26] Mills F, Swift SJ (2015). What can be gained through peer supervision?. Educ Child Psychol.

[27] (2024). Les check-lists pour la sécurité du patient. Haute Autorité de Santé.

[28] Pittenger AL, Chapman SA, Frail CK, Moon JY, Undeberg MR, Orzoff JH (2016). Entrustable professional activities for pharmacy practice. Am J Pharm Educ.

[29] Eudaley ST, Brooks SP, Jones MJ, Franks AS, Dabbs WS, Chamberlin SM (2022). Evaluation of student-perceived growth in entrustable professional activities after involvement in a transitions-of-care process within an adult medicine advanced pharmacy practice experience. Curr Pharm Teach Learn.

[30] Perrier Q, Vitale E, Tanty A (2024). Formation pratiques des étudiants en pharmacie aux soins pharmaceutiques hospitaliers. Le Pharm Clin.

[31] Siraj HH, Salam A, Azmina H (2013). Self awareness and reflective skills in the promotion of personal and professional development of future medical professionals. Educ Med J.

[32] Newsom LC, Dupree LH, Thurston MM, Vivian Liao T, Nwaesei AS (2023). A scoping review of student pharmacist-led transitions-of-care initiatives. Am J Pharm Educ.

[33] Yailian AL, Reallon E, Paillet C, Janoly-Dumenil A (2023). Analyse des pratiques de conciliation médicamenteuse auprès des prescripteurs et étudiants en pharmacie. Le Pharm Clin.

[34] Jacquet M, Maillart C, Georges F, Poumay M (2014). De la satisfaction à la performance: dépasser les indicateurs de satisfaction pour évaluer et réguler efficacement. Educ Form.

[35] Haines ST, Pittenger AL, Stolte SK (2017). Core entrustable professional activities for new pharmacy graduates. Am J Pharm Educ.

[36] Long J, Yuan MJ, Poonawala R (2016). An observational study to evaluate the usability and intent to adopt an artificial intelligence-powered medication reconciliation tool. Interact J Med Res.

