# Supplementary material for: Effect of Pharmacy Student Peer Supervision on the Accuracy of Admission Medication Reconciliation: Prospective Pre-Post Observational Study
Source: JMIR Hum Factors. 2026 Mar 9;13:e77486. doi: 10.2196/77486 (PMC12976858; doi:10.2196/77486)
Supplement: Multimedia Appendix 1 [file humanfactors-v13-e77486-s001.docx]

Appendix 1: Development of the organizational model.

**Methods**

At the start of the development phase, the initiating investigators (a pharmacist and a pharmacy resident) held a series of collaborative meetings and analyzed the data from three questionnaires. The questionnaires were designed in consultation with the pharmacists who conducted reconciliations in our institution and included both closed questions and open-ended questions.

The following elements were documented, in chronological order:

- The first questionnaire (Q1) was e-mailed to 29 clinical pharmacists and pharmacy residents between December 23rd, 2023, and January 20^th^, 2024. The purpose of this 12-item questionnaire was to gather opinions of the project and identify potential improvements in the medication reconciliation process. Respondents were asked to provide one or more keywords that represented their views on the organizational model with student peer supervision of admission medication reconciliations.
- Between January 8^th^, 2024, and January 23^rd^, 2024, the second questionnaire (Q2) was e-mailed to 30 final-year pharmacy students who conducted admission medication reconciliations after having been trained. The 9-item questionnaire gathered data on the students’ experiences with medication reconciliation, their practices, any challenges encountered, the perceived added value, and their opinions on a potential supervisory role. The questionnaire was also designed to identify the skills to be developed during the students’ training.
- The third questionnaire (Q3) was e-mailed to the same 29 clinical pharmacists and pharmacy residents between March 18^th^, 2024, and April 12^th^, 2024. It featured 20 specific scenarios related to medication reconciliation; respondents were asked to indicate whether a given task should be performed by the student or by the supervising pharmacist/resident. The evaluation criterion (consensus on task allocation) was met when the majority of the respondents categorized the scenario in the same way (i.e. as either student task or a pharmacist/resident task). The proportion of scenarios meeting the 50% agreement threshold was then calculated.

The three questionnaires were conducted using the online data collection tool Microsoft Forms® (2023 version; Microsoft Corporation, Redmond, WA, USA).

We used the survey data to develop a medication reconciliation checklist for the student peer supervision of admission medication reconciliations. Based on the scenarios outlined in Q3, the checklist was systematically applied to optimize pharmacist engagement during medication reconciliations. The checklist's primary functions include the standardization of practices, the provision of an exhaustive aide-memoire, use as a training tool, the enhancement of quality assurance, and risk mitigation.

**Results**

The questionnaire development phase involved four clinical pharmacists and a pharmacy resident

|  | Tools | Personnel involved |
| --- | --- | --- |
| Development phase | Consultation meetings | Four clinical pharmacists and one pharmacy resident |
|  | Questionnaire on the pharmacists’ feedback (Q1) | 22 clinical pharmacists replied |
|  | Questionnaire on the students’ feedback (Q2) | 20 students replied |
|  | Questionnaire on the 20 scenarios (Q3) | 17 clinical pharmacists replied |

Twenty-two (75.9%) of the 29 recipients of Q1 replied immediately or after having received a reminder. Eleven (50%) of the 22 respondents were senior pharmacists. Twenty (66.7%) of the 30 recipients of Q2 replied. Most of the pharmacists (n=15, 70%) and students (n=12, 60%) favored peer validation (Appendix 2).

Seventeen 58.6% of the 29 recipients of Q3 replied. The pharmacists felt that consultation was unnecessary in 75% of cases. This was especially true when the best possible medication history (BPMH) included less than five low-risk medications or when questions arose about a medication's presence in the hospital drug formulary.

Pharmacists primarily viewed consultation as essential for selecting alternatives to treatments not listed in the hospital drug formulary. This was particularly true when neither the pharmacy nor the patient could be consulted and when conflicted data had been obtained from different sources (Appendix 3).

The results for Q3 led to the development of a list (Appendix 4) of points to be checked by the supervising student before he/she submits the results of the reconciliation to a pharmacist for validation. Consensus on the checklist among the consulted pharmacists was reached on April 22^nd^, 2024. The checklist served as a guide for students supervisors during part of the evaluation phase.
