# Supplementary material for: Effect of Pharmacy Student Peer Supervision on the Accuracy of Admission Medication Reconciliation: Prospective Pre-Post Observational Study
Source: JMIR Hum Factors. 2026 Mar 9;13:e77486. doi: 10.2196/77486 (PMC12976858; doi:10.2196/77486)
Supplement: Multimedia Appendix 2 [file humanfactors-v13-e77486-s002.docx]

Appendix 2: Responses from pharmacists (Q1) and pharmacy students (Q2)

|  | Pharmacists' responses to Q1  n=22 | Students' responses to Q2  n=20 |
| --- | --- | --- |
| More than 5 requests per week | 14 (65) | 8 (40) |
| Number of requests per day  1  2 to 4  More than 5 | 7 (30)  12 (55)  3 (15) | 13 (65)  7 (35)  0 (0) |
| Do you encounter reconciliations that could be validated without input from a pharmacist?  No  Yes | 7 (30)  15 (70) | 10 (50)  10 (50) |
| Do you think it is feasible for a student to pre-validate a reconciliation conducted by another student?  No  Yes | 7 (30)  15 (70) | 8 (40)  12 (60) |
| Could a student be responsible for answering certain questions related to medication reconciliation, in a supervisory/support role?  No  Yes | 9 (40)  13 (60) | 8 (40)  12 (60) |
| Estimated number of errors made by students per reconciliation  1 to 3  4 to 5  More than 5 | 20 (90)  1 (5)  1 (5) |  |
| Time needed to validate a reconciliation (from the discussion with the student to the correction of discrepancies)  Less than 10 minutes  10 to 30 minutes  More than 30 minutes | 0 (0)  17 (75)  5 (25) |  |
| Do you consider that enough staff are available to conduct reconciliations?  No  Yes | 6 (25)  16 (75) |  |

Values are expressed as n (%).
