# Supplementary material for: Effect of Pharmacy Student Peer Supervision on the Accuracy of Admission Medication Reconciliation: Prospective Pre-Post Observational Study
Source: JMIR Hum Factors. 2026 Mar 9;13:e77486. doi: 10.2196/77486 (PMC12976858; doi:10.2196/77486)
Supplement: Multimedia Appendix 3 [file humanfactors-v13-e77486-s003.docx]

Appendix 3: Votes on validation in various reconciliation scenarios

| Specific scenarios in medication reconciliation | Validation by a pharmacist is essential | Validation by a trained student supervisor is possible |
| --- | --- | --- |
| Missing data on the patient’s weight and height | 1 (6) | 16 (94) |
| Chronic treatment with less than 5 common medications in a patient not self-medicating. | 3 (18) | 14 (82) |
| The student is not sure whether the drug is listed in the hospital’s central pharmacy. | 3 (18) | 14 (82) |
| The student does not identify any discrepancies at the end of the reconciliation. | 3 (18) | 14 (82) |
| None of the patient's treatments are associated with a high-risk of adverse drug events. | 3 (18) | 14 (82) |
| The reconciliation is performed in line with guidelines and training. | 3 (18) | 14 (82) |
| Estimation of the number of doses of on-demand medication. | 3 (18) | 14 (82) |
| Difficulties in retrieving information on vaccinations. | 3 (18) | 14 (82) |
| The student cannot locate the three sources of information (the patient’s records, prescriptions, and an interview with the community pharmacist) | 4 (23) | 13 (77) |
| The formulation is not specified on the prescription or by another source. | 5 (29) | 12 (71) |
| Missing data on the patient’s allergies. | 5 (29) | 12 (71) |
| Missing data on the injection or other administration of long-term treatments. | 5 (29) | 12 (71) |
| Prioritization of several reconciliations | 5 (29) | 12 (71) |
| No reply from the patient’s community pharmacy. | 6 (35) | 11 (65) |
| Missing data on adherence. | 7 (41) | 10 (59) |
| Missing data on the medication history. | 9 (53) | 8 (47) |
| Missing data on the dosing regimen. | 10 (59) | 7 (41) |
| Neither the patient’s community pharmacy nor the patient can be questioned. | 10 (59) | 7 (41) |
| When it is necessary to choose an equivalent medication from the drug formulary and set its dose level/dosing regimen. | 13 (76) | 4 (24) |
| Contradictory information is given by two or more sources | 13 (76) | 4 (24) |

Values are expressed as n (%).
