# Supplementary material for: Effect of Pharmacy Student Peer Supervision on the Accuracy of Admission Medication Reconciliation: Prospective Pre-Post Observational Study
Source: JMIR Hum Factors. 2026 Mar 9;13:e77486. doi: 10.2196/77486 (PMC12976858; doi:10.2196/77486)
Supplement: Multimedia Appendix 4 [file humanfactors-v13-e77486-s004.docx]

Appendix 4: The student supervisor's checklist

**SUPERVISION OF ADMISSION MEDICATION RECONCILIATIONS**

**Points to be checked by the student supervisor**

|  | Yes | To be revised |
| --- | --- | --- |
| Sources used for reconciliation | | |
| Medical records consulted (the reason for hospital admission, the medical history, and known treatments prior to hospital admission) |  |  |
| Presence of information from at least three different sources (including the patient’s community pharmacy and prescription data) |  |  |
| The patient’s community pharmacy: name + full postal address + phone number |  |  |
| Prescription(s) over the last 3 months: date (number of renewals) + prescriber (primary care physician or specialist) |  |  |
| Name, type (nurse/family) + phone number of caregivers, if the patient does not manage his/her medications. |  |  |
| Contact with the nursing home (for institutionalized patients) |  |  |
| If the patient has been transferred: the transfer letter and (if applicable) transfer prescriptions |  |  |
| Self-medication or off-label use: check the medications brought from home by the patient and stored securely in the care unit or covered by a prescription for self-treatment |  |  |
| Medication management | | |
| Who manages medications and/or picks them up at the community pharmacy: the patient, a caregiver, or a nurse? |  |  |
| If a pill organizer used? If so, it is filled daily or weekly? |  |  |
| Does the patient have difficult taking medications? If so, specify them. |  |  |
| Adherence has been rated on the Girerd scale. If there are adherence problems, specify them. |  |  |
| Is medication administration supervised by a nurse/caregiver? |  |  |
| In the “treatments” paragraph, the following should be mentioned for each drug: | | |
| The BRAND NAME in capital letters, the international nonproprietary name, the pharmaceutical formulation, dose level, units, and full dosing regimen (with the time of administration precisely specified) |  |  |
| If the patient is treated with a combination of several drugs from the same class: the correct drug at the correct dose level, in the correct order. |  |  |
| Frequency of administration for “as needed” (on-demand) medications |  |  |
| Verification of the absence of discrepancies between the primary care prescription and home administration (by the patient/caregiver/nurse) |  |  |
| For drugs that are not taken daily, check the date of the last administration and/or the next planned administration (e.g., vitamin D, low-molecular-weight heparin, methotrexate, bisphosphonate, bone resorption inhibitors, chemotherapeutics, etc.) |  |  |
| For eye drops: the number of drops and in which eye(s) |  |  |
| For a nasal spray: the number of sprays and in which nostril(s) |  |  |
| For ear drops: the number of drops and in which ear(s) |  |  |
| Verification of laboratory criteria for certain medications (e.g. levothyroxine, vitamin D, potassium, etc.) |  |  |
| For creams, gels and ointments: application site |  |  |
| Identification of medications not available in the central pharmacy’s formulary and not brought to the hospital by the patient: write "*not available in hospital drug formulary: drug switch suggested*" at the end of the entry  Confirm any suggested switches with the pharmacist. |  |  |
| Check that the entries in the BPMH are ordered by disease area (i.e. cardiovascular drugs are grouped together, neurological drugs are grouped together, etc.) |  |  |
| Comparison of the BPMH with the admission prescription | | |
| Compare the BPMH with prescriptions from at least the last 3 months |  |  |
| Search for and highlight discrepancies in medications/dose levels/dosing regimen |  |  |
| If the originator pharmaceutical specialty includes several active compounds available in the formulary: specify the equivalent drugs available at Lille University Medical Center. |  |  |
| Difficulties in taking medication (e.g. swallowing disorders) | | |
| Swallowing disorders present: yes or no |  |  |
| Check whether pills/tablets are crushed and/or capsules are opened. If so, which ones? |  |  |
| Check administration routes and methods:   - oral, nasogastric tube, percutaneous endoscopic gastrostomy, etc. - mixing with food and/or drinks; if yes, which ones? - medications taken together or separately? |  |  |
| If pills/tablets are crushed, has this been validated in accordance with the procedure currently in force? |  |  |
| If the patient had difficulties in taking medication, suggest other pharmaceutical formulations (to be confirmed with the pharmacist) |  |  |
| If patient has a disability, loss of autonomy, or a degenerative or disabling disease: check the ability to self-administer medications (opening boxes, removing medications from blister packs, applying topical medications, etc.) |  |  |
| Check that aerosols are being used properly |  |  |
| Any other difficulties? |  |  |
| Antibiotic therapy in the previous 3 months |  |  |
| Antibiotic therapy in the previous 3 months: yes or no? |  |  |
| Brand name + international nonproprietary name + dose level + unit + administration route + dosing regimen |  |  |
| Date and duration of prescription |  |  |
| Self-medication | | |
| Self-medication : yes or no? |  |  |
| Brand name + international nonproprietary name + dose level + pharmaceutical formulation + frequency of administration + date of last administration |  |  |
| Allergy/side effects | | |
| Allergy/side effects: yes or no? |  |  |
| Medications involved + associated symptoms and documentation (if an allergy) |  |  |
| Vaccinations | | |
| Seasonal flu and COVID vaccines: date of last vaccination, number of doses, and any history of infection (including the date of the last infection) |  |  |
| Tetanus, diphtheria, and acellular pertussis vaccine and *Pneumococcus* vaccine: date of the last vaccination |  |  |
