# Supplementary material for: Effect of Pharmacy Student Peer Supervision on the Accuracy of Admission Medication Reconciliation: Prospective Pre-Post Observational Study
Source: JMIR Hum Factors. 2026 Mar 9;13:e77486. doi: 10.2196/77486 (PMC12976858; doi:10.2196/77486)
Supplement: Multimedia Appendix 5 [file humanfactors-v13-e77486-s005.docx]

Appendix 5: Type of errors found in reconciliations supervised by students

| **Type of errors** | **n=143** |
| --- | --- |
| Frequency of administration of on-demand medication not specified | 25 (17) |
| Documented intentional divergence classified as unintentional | 13 (9) |
| Time of administration not specified | 11 (8) |
| Error regarding the availability of the molecule in the formulary | 8 (6) |
| Dose level not specified | 8 (6) |
| Dosing regimen error | 8 (6) |
| Omission of a drug | 7 (5) |
| Date of last administration of nondaily medication not mentioned | 7 (5) |
| Incomplete list of sources | 6 (4) |
| Recent antibiotic therapy not mentioned | 5 (3) |
| Error regarding the pharmaceutical formulation | 5 (3) |
| Error regarding vaccines | 5 (3) |
| Choice of an equivalent drug | 4 (3) |
| Error regarding the crushability of a medication | 4 (3) |
| Misinterpretation of adherence according to the Girerd score | 3 (2) |
| Error regarding dietary supplements | 1 (1) |
| Unknown type | 23 (16) |

Values are expressed as n (%).
