# Supplementary material for: Effect of Pharmacy Student Peer Supervision on the Accuracy of Admission Medication Reconciliation: Prospective Pre-Post Observational Study
Source: JMIR Hum Factors. 2026 Mar 9;13:e77486. doi: 10.2196/77486 (PMC12976858; doi:10.2196/77486)
Supplement: Multimedia Appendix 6 [file humanfactors-v13-e77486-s006.docx]

Appendix 6: Drug classes involved in errors in reconciliations supervised by students

| **Drug class** | **ATC System** | **n =143** |
| --- | --- | --- |
| Other analgesics and antipyretics | N02B | 28 (20) |
| Antihypertensives | C02 | 18 (13) |
| Ophthalmologicals | S01 | 17 (12) |
| Vitamins | A11 | 14 (10) |
| Antithrombotic agents | B01A | 12 (8) |
| Antibacterials for systemic use | J01 | 9 (6) |
| Drugs for constipation | A06A | 8 (6) |
| Antipsychotics | N05A | 6 (4) |
| Antiacids | A02B | 5 (3.5) |
| Blood glucose lowering drugs, excl. insulins | A10B | 5 (3.5) |
| Insulins and analogues | A10A | 4 (3) |
| Drugs for obstructive airway diseases | R03 | 4 (3) |
| Topical products for joint and muscular pain | M02 | 4 (3) |
| Lipid modifying agents | C10 | 3 (2) |
| Opioids | N02A | 2 (1) |
| Antigout preparations | M04A | 2 (1) |
| Antineoplastic agents | L01 | 2 (1) |

Values are expressed as n (%)
